# Case Report: Orthognathic surgery combined with distraction osteogenesis for severe obstructive sleep apnea secondary to unilateral temporomandibular joint ankylosis

**DOI:** 10.3389/fbioe.2026.1892710

**Published:** 2026-09-21

**Authors:** Zhengyang Wang, Zhuoga Baima, Kan Li, Siyong Gao, Huanzhong Ji, Jingyuan Li, Tianyu Zhao, Xiang Li, Guangsen Zheng

**Affiliations:** Department of Oral and Maxillofacial Surgery, Guangdong Provincial Key Laboratory of Stomatology, Guanghua School of Stomatology, Sun Yat-sen University, Guangzhou, Guangdong, China

**Keywords:** distraction osteogenesis, joint ankylosis, micrognathism, obstructive sleep apnea, orthognathic surgery

## Abstract

This case report evaluates the efficacy of combined orthognathic surgery, distraction osteogenesis, and temporomandibular joint surgery for severe obstructive sleep apnea secondary to temporomandibular joint ankylosis. A 33-year-old female presented with right temporomandibular joint ankylosis, limited mouth opening, mandibular retrusion, facial asymmetry, severe OSA (AHI: 94 events/hour, LSaO_2_: 66%), and chronic cardiac insufficiency. Treatment included distraction osteogenesis with temporomandibular joint arthroplasty to improve mouth opening, facial symmetry, and airway dimensions, followed by bimaxillary orthognathic surgery (Le Fort I osteotomy and genioplasty) to optimize occlusion, facial esthetics, and airway patency. Outcomes were assessed using cephalometric analysis, airway analysis, and CBCT imaging. Significant improvement in mandibular retrusion (ANB angle) and facial symmetry was achieved. Occlusion, airway parameters (volume, AHI, oxygen saturation), and mouth opening improved over a 24-month treatment period. Vertical distraction osteogenesis via ramus osteotomy simultaneously increased ramus height and provided condylar reconstruction. Concurrent genioplasty contributed to the expansion of the pharyngeal airway and upper airway volume. This case demonstrates that a staged protocol combining orthognathic surgery, temporomandibular joint surgery, and distraction osteogenesis can yield favorable outcomes for severe OSA secondary to temporomandibular joint ankylosis. Validation in larger cohorts with longer follow-up is required.

## 1 Background

Limited mouth opening is the cardinal clinical manifestation of temporomandibular joint ankylosis (TMJA), severely impairing mastication, speech, and oral hygiene (Xia et al., 2019; Sporniak-Tutak et al., 2011). When ankylosis occurs during childhood, it may lead to severe secondary dentofacial deformities (Jiang et al., 2018). Depending on whether the ankylosis is unilateral or bilateral, patients present with facial asymmetry, micrognathia or both (Abreu Silva et al., 2021). Dentofacial deformities secondary to unilateral TMJA commonly manifest as dental crowding, occlusal plane canting, midline deviation, and significant mandibular retrusion, resulting in facial asymmetry with a bird-face appearance (Jung et al., 2017; Fariña et al., 2018).

Concurrent micrognathia compromises facial esthetics, occlusion, and oropharyngeal airway volume, potentially causing obstructive sleep apnea (OSA) (Yadav et al., 2021). Given the coexistence of TMJA, dentofacial deformity, and OSA, treatment objectives must be clearly defined and surgical interventions appropriately sequenced. However, the optimal order for releasing ankylosis and correcting maxillomandibular deformities remains controversial (Zhu et al., 2013; Bi et al., 2020). While relieving limited mouth opening has traditionally been the primary goal (Fariña et al., 2018), severe mandibular retrusion accompanied by airway stenosis and sleep-related hypoxemia may necessitate prioritizing OSA correction (Andrade et al., 2012; Chaudhary et al., 2024).

Recently, combined treatment protocols integrating orthognathic surgery, TMJ surgery, and distraction osteogenesis (DO) have shown favorable outcomes in managing severe OSA secondary to TMJA (Zhang et al., 2018). Preoperative airway assessment enables clinicians to evaluate the severity and location of stenosis, thereby facilitating optimal surgical planning and staging (Shaeran and Samsudin, 2019; Liu et al., 2017).

This case report describes a 33-year-old female with unilateral TMJA and resultant mandibular retrusion who had experienced limited mouth opening, snoring, nocturnal arousal due to breathlessness, and daytime somnolence for 30 years. The combined protocol achieved significant improvements in facial esthetics, occlusion, mouth opening, and airway patency. This case demonstrates that an airway-focused approach, guided by comprehensive preoperative airway and facial assessments and utilizing DO, TMJ surgery, and orthognathic surgery, can rapidly increase airway volume while concurrently addressing mouth opening limitation, occlusal disorders, and facial esthetics.

## 2 Case presentation

A 33-year-old female presented with a 30-year history of mandibular retrusion with facial asymmetry, limited mouth opening, severe snoring, nocturnal arousal due to breathlessness, and daytime somnolence. Polysomnography (PSG) revealed an apnea-hypopnea index (AHI) of 94 events/hour and a lowest oxygen saturation (LSaO_2_) of 66%, indicating severe OSA with marked hypoxemia. Prior evaluation had documented chronic heart failure, type II chronic respiratory failure, and hypokalemia. The patient denied any history of craniofacial trauma.

Extraoral examination revealed marked facial asymmetry with the gnathion deviated approximately 15 mm to the right, a shortened lower facial third, and right-elevated maxillary occlusal plane canting. A lateral view demonstrated severe mandibular and chin retrusion, presenting a bird-face appearance. Maximum interincisal opening was 0 cm. Bilateral temporomandibular joints exhibited no mobility, with neither spontaneous nor palpation-induced pain. Intraoral examination revealed that the maxillary and mandibular central incisor midlines were deviated 5 mm and 10 mm to the right, respectively. Overjet was 17 mm, and overbite was 9 mm (Figure 1A). Cephalometric measurements were as follows: ∠SNA 76.03°, ∠SNB 60.96°, ∠ANB 15.07°, ∠OP-SN 27.34°, ∠U1-SN 102.64°, ∠L1-MP 126.51°, and ∠MP-SN 47.05° (Supplementary Figure S1). The panoramic radiograph revealed abnormal right TMJ morphology (Figures 2A–C). CBCT demonstrated suspected right TMJ ankylosis, altered left TMJ space, and severe mandibular retrusion with facial asymmetry (Figures 2D–G). Methodological details regarding the imaging protocol and airway analysis are provided in the Supplementary Material.

**FIGURE 1:**
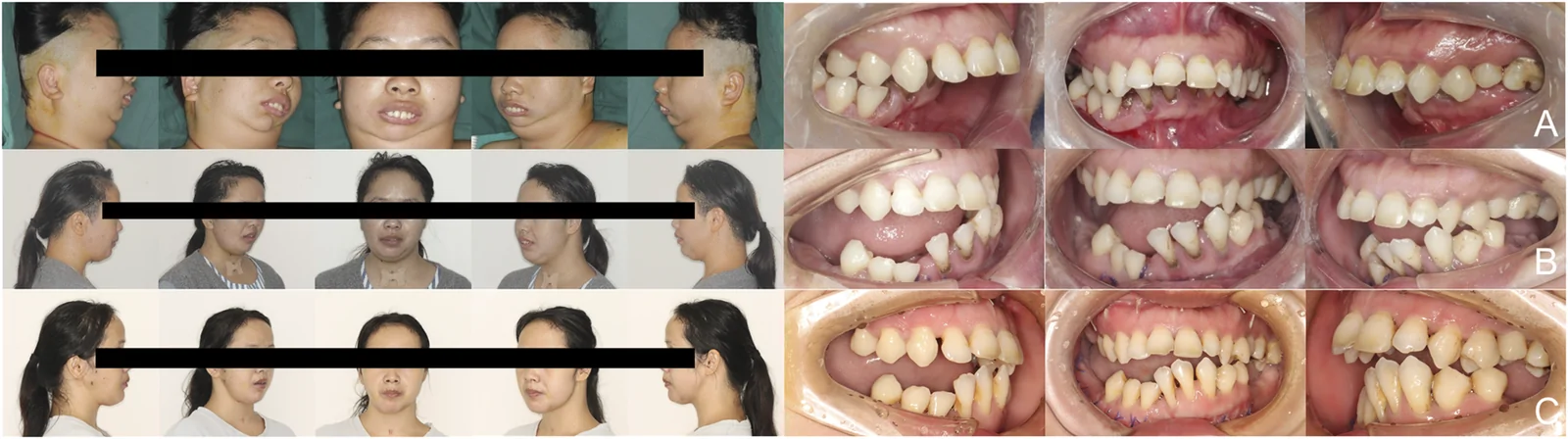
Facial and intraoral photographs **(A)** Pretreatment **(B)** Post-first-stage treatment **(C)** Post-second-stage treatment.

**FIGURE 2:**
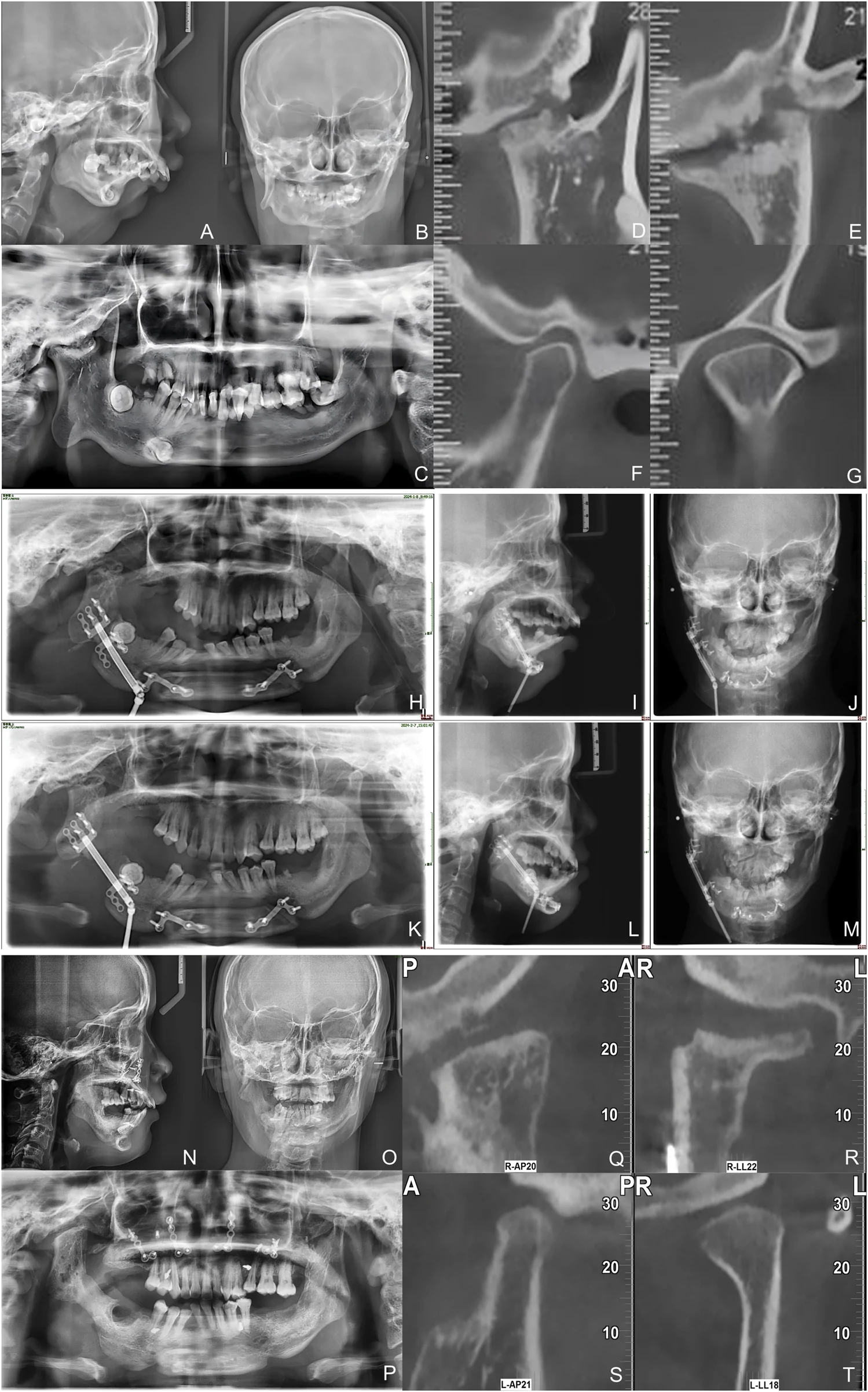
Radiographic findings (**A-C**) Pretreatment lateral cephalogram, frontal cephalogram, and panoramic radiograph (**D-G**) Pretreatment TMJ cross-section radiographs (**H**–**J**) Panoramic radiograph, lateral cephalogram, and frontal cephalogram at 1 week post-first-stage surgery (**K**–**M**) Panoramic radiograph, lateral cephalogram, and frontal cephalogram at 1 month post-first-stage surgery (**N**–**P**) Post-second-stage treatment lateral cephalogram, frontal cephalogram, and panoramic radiograph (**Q**–**T**) Post-second-stage treatment TMJ cross-section radiographs.

### 2.1 Diagnoses

(1) Severe OSA; (2) Severe sleep-related hypoxemia; (3) Right TMJA; (4) Mandibular retrusion; (5) Facial asymmetry; (6) Retrognathia; (7) Left TMJ disorder (osteoarthritis); (8) Suspected hypertension; (9) Suspected chronic heart failure.

### 2.2 Treatment plan

Given the severity of sleep-related hypoxemia, OSA, and limited mouth opening, treatment planning was guided by clinical urgency according to the following priority hierarchy: airway improvement > restoration of mouth opening > occlusal correction > facial esthetics optimization. Each stage was designed based on radiographic findings, digital simulation and reconstruction, subjective symptoms, and the patient’s chief concerns to address the primary problem at that time point. Comprehensive orthodontic treatment was recommended but declined by the patient for financial reasons. The treatment plan was therefore designed as a surgery-only protocol. Surgical planning was performed using Dolphin Imaging software, combining cephalometric analysis with CBCT-based virtual surgical simulation.

The first stage prioritized functional improvement and quality of life. Given the urgency of airway management and restoration of mouth opening, the plan focused on releasing the ankylosed joint and performing DO with genioplasty to expand the airway, without requiring precise restoration of facial esthetics or occlusion at this stage. The second stage, based on comprehensive digital orthognathic surgical planning, utilized digitally fabricated surgical guides to optimize facial harmony and occlusal stability.

### 2.3 First-stage treatment

A submandibular approach with layered dissection was employed to preserve the marginal mandibular nerve. After subperiosteal elevation, the right mandibular ramus was vertically osteotomized while maintaining inferior alveolar neurovascular integrity. A single-vector intraoral distractor (CMF Retractor, CIBEI) was secured along the planned distraction axis. Intraoperative activation (2–3 mm) confirmed proper vector alignment. The temporalis muscle attachments were stripped upward bilaterally along the mandibular ramus, and a reciprocating saw was used to resect the bilateral coronoid processes at the level of the sigmoid notch. Advancement genioplasty of 7 mm was performed to ameliorate retrognathia.

An incision was made along the anterior border of the right ear, with meticulous dissection to safeguard the zygomatic and temporal branches of the facial nerve. The periosteum and joint capsule were incised at the anterior external auditory canal margin to expose the ankylosed TMJ, where complete loss of the articular disc and joint space was observed alongside focal bony hypertrophy and sclerosis. The abnormal condylar mass and hyperplastic bone were excised with a reciprocating saw and surgical bur, followed by articular surface contouring via grinding. A Dacron patch was trimmed and interposed between the articular surfaces. The wounds were closed primarily in layers (Figures 3A–C).

**FIGURE 3:**
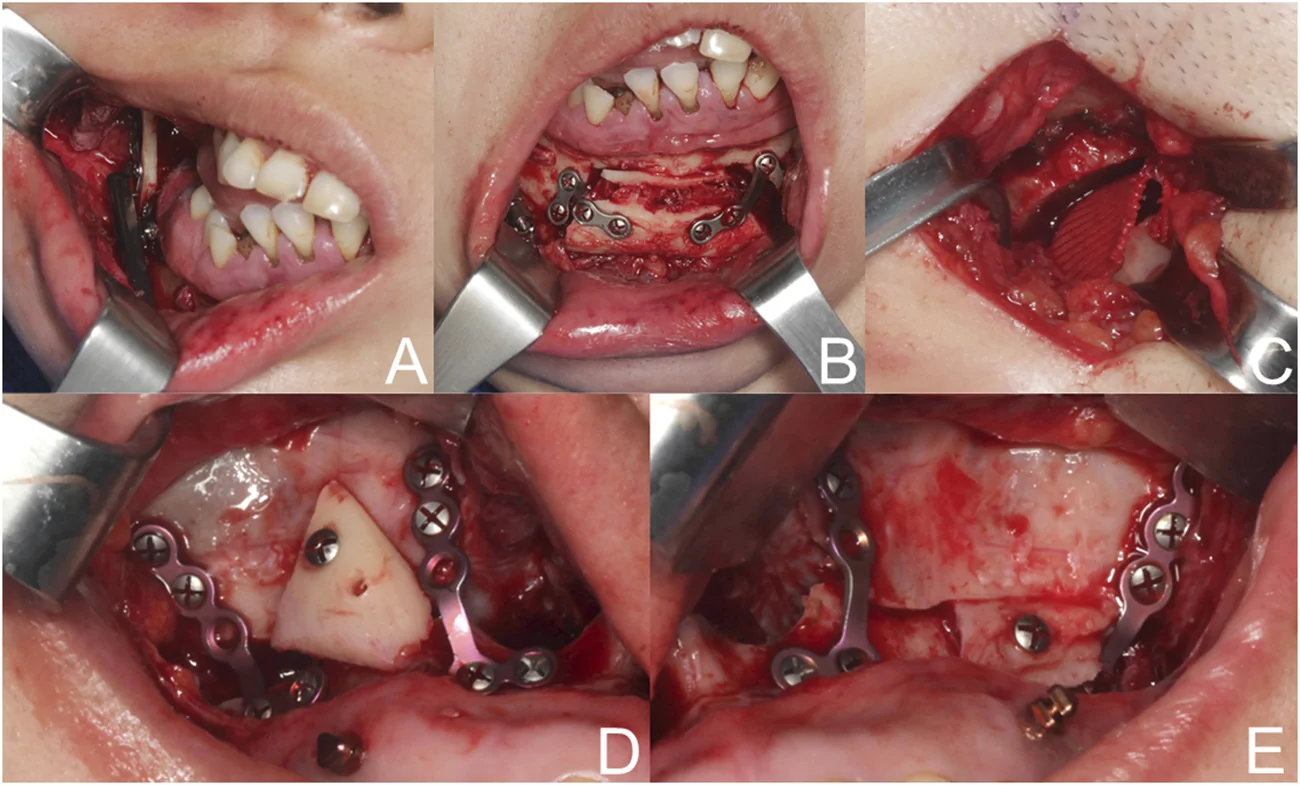
Representative intraoperative photos **(A)** Distraction osteogenesis **(B)** Genioplasty **(C)** Right temporomandibular joint arthroplasty **(D) (E)** Le fort I osteotomy.

After a 7-day latency period, distraction commenced at 0.4 mm twice daily. The consolidation phase was maintained for 6 months, with device removal following radiographic confirmation of regenerate mineralization (Figures 2H–M; Supplementary Figure S2). Postoperatively, mouth opening, occlusion, and facial esthetics were improved (Figure 1B).

### 2.4 Second-stage treatment

Seven months after the first-stage treatment, the right mandibular distractor was removed. Four VectorTAS miniscrews (Ormco Corp., Glendora, CA, United States) were inserted into the maxilla and mandible. The patient then underwent a bilateral Le Fort I osteotomy. A final occlusal splint was placed, and wire intermaxillary fixation (IMF) was applied to temporarily secure the planned occlusal relationship, allowing the maxilla to be positioned as planned. On each side, two L-shaped titanium plates were placed and fixed with eight titanium screws (Synthes, Switzerland) (Figures 3D,E).

To further improve facial esthetics, a small amount of bone was harvested from the right mandibular body using a reciprocating saw (Medicom surgery®). The harvested bone block was shaped to an appropriate size and implanted bilaterally at the lateral margins of the pyriform apertures to augment paranasal fullness. The bilateral alar bases were then suture-ligated intraorally to narrow alar width. Radiographic analysis was performed at each stage throughout the treatment process.

Post-surgical follow-up assessments included airway analysis, cephalometric measurements, CBCT imaging, and clinical occlusal and facial evaluation.

### 2.5 Treatment results

At 24 months, facial symmetry was substantially restored, with an approximately horizontal occlusal plane, proportionate facial thirds, and a centered gnathion. The lateral view showed a straight facial profile (Figure 1C). The outcome of DO was satisfactory, as demonstrated by adequate bone volume and near-normal bone density on radiographic evaluation (Figures 2N–P). Maximal interincisal opening was 24 mm with a straight opening pattern (“↓”) (Supplementary Figure S3). No TMJ clicking was palpable bilaterally, and no spontaneous pain or tenderness to palpation was noted in the bilateral preauricular regions. Radiographs demonstrated a clear joint space and regular, sclerotic bone margins, indicating successful remodeling and functional recovery (Figures 2Q–T). PSG showed AHI reduction from 94 to 5.83 events/hour and LSaO_2_ improvement from 66% to 88%, consistent with mild OSA and mild hypoxemia. Preoperative and 6-month postoperative airway parameters are presented in Table 1 and Figure 4. A concise timeline of the diagnostic and treatment course is summarized in Supplementary Table S1.

**TABLE 1:** Changes in airway volume and morphology before surgery and six months postoperatively.

Time point	Total airway volume (mm^3^)	Total airway length (mm)	Mean airway cross-sectional area (mm^2^)
Pre-DO	4,002	47.9	154
Six months Post-DO	11,545	46.4	290.5

**FIGURE 4:**
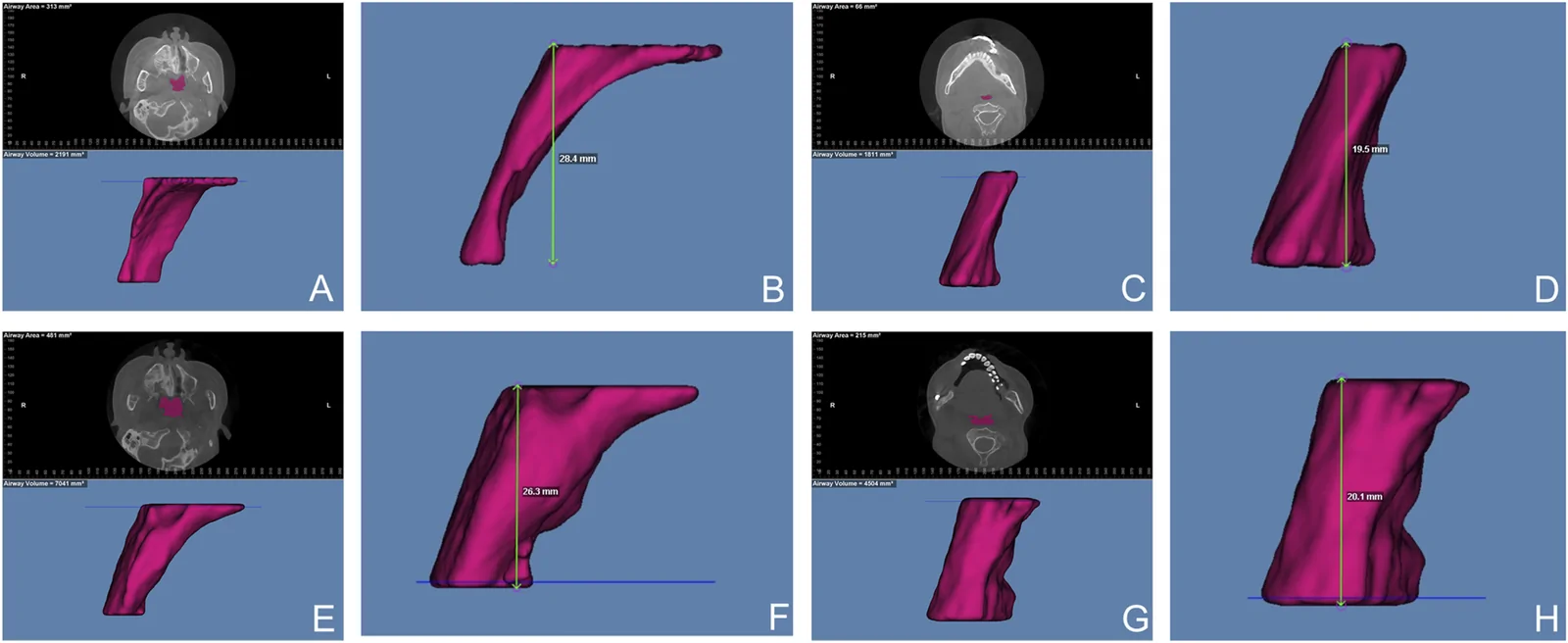
Changes in airway volume before and 6 months after primary treatment **(A–D)** Pre-first-stage airway imaging **(E–H)** 6 months post-first-stage airway imaging **(A,E)** Retropharyngeal airway superior area **(B,F)** Retropharyngeal airway morphology **(C,G)** Retrolingual airway superior area **(D,H)** Retrolingual airway morphology.

## 3 Discussion

### 3.1 Mechanisms of OSA secondary to TMJA

TMJA is characterized by loss of joint mobility resulting from trauma, inflammation, or surgical intervention (Xia et al., 2019; Bello et al., 2012). As the condyle is the primary mandibular growth center, intra-articular ankylosis occurring during childhood can lead to developmental deformities of the lower face (Zhu et al., 2011). Furthermore, impaired masticatory function causes micrognathia characterized by mandibular retrusion and facial asymmetry to worsens progressively with age (Yang et al., 2011). Although improved early management of condylar fractures has reduced TMJA-related dentofacial deformities in developed countries, such deformities remain prevalent in remote regions of developing nations (Bello et al., 2012). Studies have demonstrated that earlier TMJA onset correlates with more severe micrognathia, and affected patients exhibit extremely narrow upper airways predisposing them to severe OSA (Liu et al., 2016). Additional research suggests that the airway characteristics of these patients are associated with a high prevalence of OSA, reflecting a transmural pressure gradient in which intraluminal pressure at the collapse site falls below extraluminal pressure (Fariña et al., 2018). Compared with patients without OSA, patients with micrognathia secondary to unilateral TMJA have similar hypopharyngeal volumes but reduced velopharyngeal and oropharyngeal volumes (Shaeran and Samsudin, 2019; Walsh et al., 2008; Muto et al., 2008). This finding indicates that mandibular retrusion predominantly affects the velopharyngeal and oropharyngeal regions, and the reduction in velopharyngeal volume also suggests maxillary involvement beyond the mandible alone (Yew et al., 2024).

### 3.2 Diagnosis of OSA

Currently, the internationally recognized diagnostic criteria for adult OSA require fulfillment of either (A+ B) or C (Kapur et al., 2017).

1. At least one of the following: (1) complaints of sleepiness, non-restorative sleep, daytime fatigue, or insomnia symptoms; (2) nocturnal awakenings due to breathlessness, gasping, or choking; (3) habitual snoring, breathing interruptions, or both, as reported by a bed partner or roommate; (4) diagnosed hypertension, mood disorder, cognitive dysfunction, coronary artery disease, stroke, congestive heart failure, atrial fibrillation, or type 2 diabetes mellitus.
2. PSG or OCST findings: obstructive respiratory events (obstructive and mixed apneas, hypopneas, and respiratory effort-related arousals) ≥5 events/hour.
3. PSG or OCST findings: obstructive respiratory events ≥15 events/hour.

According to the current international standards, the severity grading of adult OSA is primarily based on two parameters: AHI and LSaO_2_, as detailed in Table 2.

**TABLE 2:** AASM grading criteria for adult obstructive sleep apnea and hypoxemia.

Criterion	OSA severity	Indicator	Hypoxemia severity
AHI (events/h)	​	LSaO_2_ (%)	​
5–15	Mild	85–90	Mild
15–30	Moderate	80–85	Moderate
>30	Severe	<80	Severe

### 3.3 Orthognathic surgery of OSA secondary to TMJA

Childhood-onset TMJA causes varying degrees of micrognathia due to insufficient mandibular growth, with bilateral cases demonstrating the most severe manifestations (Hsieh et al., 2016). Furthermore, patients with micrognathia frequently present with pharyngeal airway narrowing, and a subset develop severe OSA (Chan et al., 2020). In recent years, combined protocols have demonstrated favorable outcomes in the comprehensive management of severe OSA secondary to TMJA, although the optimal surgical sequence remains debated. Preoperative airway assessment helps evaluate the severity and location of stenosis, guiding surgical planning. For example, in OSA patients with severe airway stenosis (minimal cross-sectional area), restoration of airway dimensions and volume should be prioritized during mandibular reconstruction. The ultimate objectives are airway expansion, OSA resolution, and concurrent improvement of mouth opening, occlusal function, and facial harmony (Agarwal et al., 2021).

DO is the most direct and effective method for airway expansion, particularly in cases of micrognathia secondary to bilateral TMJA (Chaudhary et al., 2024; Wang et al., 2003). DO can achieve mandibular lengthening exceeding 20.0 mm, effectively correcting severe dentofacial deformities while demonstrating favorable therapeutic effects on concurrent OSA (Wu et al., 2024). Vertical DO via ramus osteotomy simultaneously increases ramus height and reconstructs the condyle with relatively stable outcomes (Shang et al., 2012; Zhang et al., 2017). For recurrent ankylosis, most investigators recommend alloplastic total joint replacement or prosthetic condylar reconstruction (Yang et al., 2018). Importantly, genioplasty performed concurrently with DO further expands the pharyngeal airway, increases upper airway volume, and improves micrognathia (Valls-Ontañón et al., 2024).

The recurrent intermittent apneas or hypopneas caused by OSA may lead to severe systemic complications, characterized by high morbidity, numerous comorbidities, and potential lethality (Yeghiazarians et al., 2021; Veasey et al., 2019). Moreover, restricted mandibular movement, limited mouth opening, malnutrition, and endocrine dysfunction in TMJA patients may exacerbate OSA severity (Chaudhary et al., 2024; Akset et al., 2023). In this case, the combination of DO with orthognathic surgery and TMJ surgery achieved favorable therapeutic outcomes in a patient with micrognathia and severe OSA secondary to unilateral TMJA.

### 3.4 Biological basis of regeneration in the combined protocol

DO may be regarded as a form of endogenous tissue engineering, in which gradual tensile strain, rather than an exogenous graft, drives new bone formation. The osteotomy hematoma recruits mesenchymal stromal cells and upregulates TGF-β and BMPs. During distraction, collagen fibers align along the distraction vector to form a fibrous interzone from which mineralization proceeds bidirectionally, a process strictly dependent on VEGF-mediated neoangiogenesis (Morcos et al., 2015). Mechanical strain is transduced into coupled osteogenic and angiogenic signaling through Integrin-FAK, TGF-β/BMP, Wnt/β-catenin, and HIF-1α pathways (Hu et al., 2024).

The protocol applied here (7-day latency, 0.4 mm twice daily, 6-month consolidation) was chosen to remain within the biologically favorable window, as excessively rapid distraction favors fibrous nonunion whereas excessively slow distraction risks premature consolidation (Morcos et al., 2015; Hu et al., 2024). Vertical ramus DO additionally regenerated a load-bearing neocondyle; because condylar adaptation is rate-dependent, with articular irregularities persisting at high distraction rates in experimental models, a moderate rate with prolonged consolidation was adopted (Thurmüller et al., 2002).

The Dacron interposition was intended to establish a fibrous, non-osteogenic interface resistant to heterotopic bone formation while permitting a functional pseudarthrosis. Because alloplastic materials carry higher reankylosis rates than autogenous alternatives, this represents a pragmatic, case-specific choice requiring continued surveillance rather than a generalizable recommendation.

### 3.5 Critical appraisal of clinical outcome

This combined protocol achieved substantial improvements: AHI decreased from 94 to 5.83 events/hour and LSaO_2_ improved from 66% to 88%, consistent with outcomes reported by Chaudhary et al. in similar populations (Chaudhary et al., 2024). Facial symmetry was effectively restored, and vertical DO successfully increased ramus height and reconstructed the condyle. No major surgical complications occurred.

The novelty of this report lies in the treatment sequencing. Published protocols either perform distraction before joint release or release the joint first and defer distraction and genioplasty to a later stage (Zhang et al., 2018; Li et al., 2012). In the present case, interpositional arthroplasty, bilateral coronoidectomy, vertical ramus distraction, and genioplasty were completed in a single operation. A preoperative AHI of 94 events/hour with heart failure and type II respiratory failure, exceeding the mean values of approximately 51–57 events/hour in published cohorts, justified this airway-first strategy (Chaudhary et al., 2024; Yadav et al., 2014).

However, several limitations should be acknowledged. The postoperative maximal interincisal opening of 24 mm, though markedly improved from complete ankylosis, remains below the 35–40 mm range typically reported after TMJA surgery, possibly reflecting insufficient physiotherapy intensity (Shetty et al., 2019). The patient declined pre- and post-operative orthodontic treatment for the second-stage surgery due to financial constraints and high satisfaction with the outcome. This introduces some uncertainty regarding the long-term stability of occlusal and facial relationships (Albayrak et al., 2025). However, this was anticipated during the preoperative Dolphin surgical planning, in which occlusion and facial esthetics were simultaneously optimized to the greatest extent possible, ensuring adequate overjet, overbite, and broad posterior occlusal contacts. The planned orthodontic-orthognathic protocol is detailed in the Supplementary Material. Additionally, residual mild OSA (AHI 5.83) and mild hypoxemia (LSaO_2_ 88%) suggest that further airway optimization may be warranted, and adjunctive therapies such as positional therapy or oral appliances should be considered if symptoms recur.

Regarding surgical sequencing, three approaches were considered: (1) a joint-first approach (prioritizing TMJ surgery, followed by DO and orthodontic-orthognathic treatment); (2) an airway-first approach (prioritizing DO, followed by TMJ surgery with concurrent orthognathic surgery and subsequent orthodontics); and (3) a simultaneous joint-and-airway approach (concurrently restoring mouth opening and correcting mandibular retrusion, followed by orthodontic-orthognathic treatment).

Regarding long-term stability, Xia et al. reported a mean resorption rate of 59.4% in ramus height following condylar reconstruction by transport DO (Xia et al., 2020), and Hassan and Mohamed observed significant skeletal relapse over 8.5 years of follow-up, although facial esthetics remained satisfactory (Hassan and Mohamed, 2019). Re-ankylosis risk, though mitigated by the Dacron interpositional patch, cannot be eliminated (Mittal et al., 2019). Regarding expected long-term effects on airway function, Camacho et al. demonstrated that while AHI improvements were well maintained in the long term (4 to <8 years), the mean AHI increased to moderate OSA levels in the very long term (≥8 years) (Camacho et al., 2019). Pottel et al. similarly reported that among patients initially cured by maxillomandibular advancement, 66.7% remained stable at a median follow-up of 19 years; however, aging and weight gain may counterbalance the positive surgical effects over time, and they recommended re-evaluation every 5 years (Pottel et al., 2019).

The overall treatment burden was considerable, requiring two major surgical stages under general anesthesia, prolonged hospitalization, and a 24-month treatment period with an indwelling distraction device during the consolidation phase. Nevertheless, this burden must be weighed against the patient’s life-threatening condition, namely, severe OSA with chronic cardiac and respiratory failure (Yadav et al., 2014; Leung and Lai, 2018).

Accordingly, long-term follow-up should include continued physiotherapy, periodic TMJ surveillance with imaging, repeat polysomnography at regular intervals, cardiovascular monitoring, and reconsideration of orthodontic treatment. Should significant relapse or re-ankylosis occur, revision procedures including alloplastic total joint replacement may be necessary (Xia et al., 2020; Mittal et al., 2019).

As with any single-patient case report, generalizability is limited. Moreover, because the patient was managed within routine clinical care, functional outcomes such as masticatory performance, speech, pain, and physiotherapy adherence were not assessed with validated instruments, and a structured patient perspective and patient-reported outcome measures were not prospectively collected and could not be reconstructed retrospectively; the subjective benefit of treatment therefore remains unquantified. Although all measurements were repeated by a single trained examiner, no formal intraobserver or interobserver reliability analysis was performed, leaving measurement error undetermined. Finally, the 24-month follow-up is insufficient to capture skeletal relapse, regenerate resorption, re-ankylosis, or late deterioration of airway parameters, and continued longitudinal surveillance is required to establish durability.

## 4 Conclusion

In this case, a staged, airway-prioritized surgical protocol combining DO, orthognathic surgery, and TMJ reconstruction was effective for managing severe OSA secondary to unilateral TMJA with micrognathia. The approach yielded marked improvements in airway dimensions, sleep parameters, mouth opening, occlusal function, and facial symmetry. Vertical DO via ramus osteotomy successfully increased ramus height and reconstructed the condyle, while simultaneous genioplasty further expanded pharyngeal volume. However, these preliminary findings require validation in larger cohorts with longer follow-up before this coordinated, multi-procedural framework can be recommended for complex dentofacial deformities associated with TMJA and severe OSA.

## Data Availability

The original contributions presented in the study are included in the article/Supplementary Material, further inquiries can be directed to the corresponding author.
