# Supplementary material for: Case Report: Orthognathic surgery combined with distraction osteogenesis for severe obstructive sleep apnea secondary to unilateral temporomandibular joint ankylosis
Source: Front Bioeng Biotechnol. 2026 Sep 21;14:1892710. doi: 10.3389/fbioe.2026.1892710 (PMC13635317; doi:10.3389/fbioe.2026.1892710)
Supplement: Supplementary file 2 [file Supplementaryfile1.docx]

# 1 Imaging Protocol and Airway Analysis

CBCT images were acquired using a NewTom VG unit (Quantitative Radiology, Verona, Italy) with the patient in a standing position. The acquisition parameters were 110 kVp, 7 mA, a 16*16 cm field of view, and an isotropic voxel size of 0.30 mm. Data were exported in DICOM format and imported into Dolphin Imaging 11.9 Premium (Dolphin Imaging & Management Solutions, Chatsworth, CA, USA). Before measurement, the head was reoriented so that the Frankfort horizontal (FH) plane was horizontal. The default window width and window level settings were retained throughout the analysis.

Airway segmentation was semi-automated. A trained examiner manually defined the upper-airway region of interest (ROI). The superior boundary followed the posterosuperior mucosal surface of the nasopharynx. The anterior boundary was a plane passing through the posterior nasal spine and perpendicular to both the FH and midsagittal planes. A plane passing through the fourth cervical vertebra (C4) formed the inferior boundary. Seed points were then placed within the airway lumen, after which the software automatically identified and segmented the connected airway space. No user-defined numerical grayscale threshold was entered or adjusted. The same boundary definitions, segmentation procedure, and software settings were applied to both preoperative and postoperative datasets. Total airway volume, airway length, and mean cross-sectional area were calculated.

All measurements were performed by a single trained examiner and were repeated twice to assess the measurement accuracy.

# 2 Planned Orthodontic Protocol and Expected Outcome

Although the patient ultimately declined orthodontic treatment, a comprehensive orthodontic-orthognathic protocol had been planned.

Following first-stage surgery and consolidation, presurgical orthodontics was planned to achieve dental decompensation prior to the Le Fort I osteotomy. Objectives included alignment and leveling of both arches, decompensation of severely proclined mandibular incisors to unmask the true skeletal discrepancy, transverse arch width coordination, correction of dental midline deviations, and leveling of the occlusal plane cant. Miniscrew anchorage was planned for vertical and sagittal control, particularly given the high mandibular plane angle.

Postsurgical orthodontics was planned to optimize interdigitation, normalize overjet and overbite, finalize midline coordination, and manage residual edentulous spaces through either orthodontic space closure or prosthetic rehabilitation as indicated.

Orthodontic treatment was expected to yield a stable, functional occlusion with enhanced long-term skeletal stability, as presurgical decompensation permits maximal surgical correction and postsurgical detailing provides occlusal locking that resists relapse (1).

# 3 Supplementary Figures

Figure S1. Pretreatment lateral cephalogram with annotated cephalometric landmarks and reference planes.

Figure S2. Pre-second-stage treatment lateral cephalogram with annotated cephalometric landmarks, reference planes, and the distraction vector.

Figure S3. Post- first-stage treatment mouth opening photograph

# 4 Supplementary Table

**Table S1. Timeline of Clinical Events**

| **Time Point** | **Event Category** | **Key Event** | **Diagnosis / Intervention / Outcome** |
| --- | --- | --- | --- |
| Age 3 (30 years prior) | Medical history | Onset of mandibular retrusion, facial asymmetry, limited mouth opening, snoring, nocturnal arousal due to breathlessness, daytime somnolence | Right TMJ ankylosis (etiology unknown; denied craniofacial trauma) |
|  | Past medical history | Diagnosis of chronic heart failure, type II chronic respiratory failure, hypokalemia |  |
| Initial presentation (age 33) | Clinical examination | Extraoral: gnathion deviated 15 mm rightward, shortened lower facial third, right-elevated occlusal plane canting, severe mandibular and chin retrusion; MIO: 0 cm; bilateral TMJs immobile, no pain | Diagnoses: severe OSA, severe sleep-related hypoxemia, right TMJA, mandibular retrusion, facial asymmetry, retrognathia, left TMJ osteoarthritis, suspected hypertension, suspected chronic heart failure |
| Initial presentation | Polysomnography | AHI: 94 events/hour; LSaO₂: 66% | Severe OSA; severe hypoxemia |
| Initial presentation | Cephalometric analysis | ∠SNA 76.03°, ∠SNB 60.96°, ∠ANB 15.07°, ∠OP-SN 27.34°, ∠U1-SN 102.64°, ∠L1-MP 126.51°, ∠MP-SN 47.05° | Severe skeletal Class II with high mandibular plane angle |
| Initial presentation | CBCT imaging & airway analysis | Suspected right TMJ ankylosis, altered left TMJ space, severe mandibular retrusion; airway volume: 4002 mm³, airway length: 47.9 mm, mean cross-sectional area: 154 mm² | Confirmed diagnoses; severe airway compromise |
| Initial presentation | Treatment planning | Staged surgery-only protocol; priority: airway improvement > mouth opening > occlusion > facial esthetics; patient declined orthodontic treatment for financial reasons | Digital surgical simulation (Dolphin Imaging) |
| First-stage surgery (2024.1, T0) | Surgical intervention | Right mandibular vertical ramus osteotomy with intraoral distractor placement; bilateral coronoidectomy; advancement genioplasty (7 mm); right TMJ interpositional arthroplasty (excision of ankylosed mass, Dacron patch placement) | Simultaneous joint release and airway expansion |
| T0 + 7 days | Distraction initiation | Distraction commenced at 0.4 mm × 2 times/day |  |
| End of distraction | Distraction completion | Active distraction completed; consolidation phase initiated |  |
| T0 + 6 months | Consolidation completed | Radiographic confirmation of regenerate mineralization; | Improved mouth opening, occlusion, and facial esthetics |
| T0 + 6 months | Postoperative airway analysis | Airway volume: 11,545 mm³, airway length: 46.4 mm, mean cross-sectional area: 290.5 mm² | ~189% increase in airway volume |
| First-stage surgery (2024.8, T0 + 7 months) | Surgical intervention | Removal of right mandibular distractor; placement of 4 VectorTAS miniscrews; bilateral Le Fort I osteotomy (L-shaped titanium plate fixation); autogenous bone graft augmentation at pyriform apertures; bilateral alar base suture ligation | Optimization of occlusion and facial esthetics |
| T0 + 24 months | Final follow-up: clinical | Facial symmetry restored, horizontal occlusal plane, proportionate facial thirds, centered gnathion, straight profile; MIO: 24 mm with straight opening pattern; no TMJ clicking, pain, or tenderness bilaterally |  |
| T0 + 24 months | Final follow-up: PSG | AHI: 5.83 events/hour; LSaO₂: 88% | Mild OSA; mild hypoxemia |
| T0 + 24 months | Final follow-up: imaging | DO regenerate: adequate bone volume and near-normal density; TMJ: clear joint space, regular sclerotic bone margins | Successful remodeling and functional recovery |

# 5 References

1.	Gao X, Wang T, Song J. Orthodontic and surgical management of a patient with severe skeletal class II deformity and facial asymmetry: a case report with a 5-year follow-up. Am J Orthod Dentofac Orthop: Off Publ Am Assoc Orthod Const Soc Am Board Orthod. 2017 Apr;151(4):779–92. doi:10.1016/j.ajodo.2016.02.034 PubMed PMID: 28364902.
